# Editorial: The contribution of family physicians to African health systems – A call for short reports

**DOI:** 10.4102/phcfm.v13i1.2925

**Published:** 2021-04-28

**Authors:** Robert Mash

**Affiliations:** 1 Division of Family Medicine and Primary Care, Faculty of Medicine and Health Sciences, Stellenbosch University, Cape Town, South Africa

A recent scoping review on family medicine in sub-Saharan Africa concluded that:

Evidence of effectiveness and impact is still limited as the discipline is reasonably young in sub-Saharan Africa, with low numbers of family physicians. Opposition due to lack of understanding remains, but the positive perceptions of key stakeholders and the motivation of family physicians, together with evidence from elsewhere, suggest that the discipline can fill a niche and potentially improve quality of care. Political will and support is pivotal and will enable the discipline to create the critical mass to place family medicine at the forefront, to reach universal health coverage and contribute to the achievement of the sustainable development goals in sub-Saharan Africa.^1^

Family physicians are found in many countries of sub-Saharan Africa, but the numbers can vary from just a handful, as in Zimbabwe,^2^ to more than a thousand in South Africa.^3^ Their location in the health system can also vary. In Nigeria, for example, they are often found in tertiary hospitals, whilst in South Africa they are more likely to be employed in health districts as part of district specialist teams, district hospitals or in primary care.^1^

As specialists in family medicine, family physicians are an expensive and highly trained part of the workforce. Some countries, such as Botswana and Malawi, have committed to having them as part of the district health system, whilst others are ambivalent. In all settings they work as part of multidisciplinary teams with doctors, nurse practitioners, clinical officers, pharmacists and others. African health systems cannot afford to have family physicians as the main primary care provider and do not have a sufficient supply for this. Doctors are a scarce resource in all countries. Some countries, such as South Africa, have seen a competency gap at small and often rural district hospitals that family physicians can fill.^4^

Their role in the health system may be that of a clinician and consultant, a capacity builder and clinical trainer or as a leader of clinical governance to improve the quality of care and patient safety.^5^ There is some evidence that they have an impact in all these roles, more so than medical officers, and it may be greater in district hospitals.^6,7^ However, as the scoping review concluded, we need more evidence of their unique contribution to African health systems.

What is their contribution to creating high-performing primary healthcare systems and district health services that can lead the way to universal health coverage and the known benefits in terms of health status, responsiveness, equity, resilience and efficiency? What do they bring that other more established cadres do not or cannot?

This editorial supports the call for short reports (https://aosis.co.za/call-for-short-reports-the-african-journal-of-primary-health-care-family-medicine-phcfm/) on the contribution of family physicians to African health systems. Many family physicians have made a significant and unique difference to service delivery in their local context but have not captured this through applied original research. This call for short reports gives family physicians the chance to tell their stories and craft a collection of more narrative evidence on how they are making a difference. We particularly encourage family physicians from a broad range of countries to use this opportunity. Please take note of the requirements in the call for short reports, and I look forward to your submissions.
